# Integrated photonic polarization synthesizer and analyzer

**DOI:** 10.1038/s41377-026-02405-3

**Published:** 2026-07-10

**Authors:** Carson G. Valdez, Anna J. Miller, Anne R. Kroo, Charles Roques-Carmes, David A. B. Miller, Olav Solgaard

**Affiliations:** Stanford University, Ginzton Laboratory, 348 Via Pueblo Mall, Stanford, CA 94305 USA

**Keywords:** Integrated optics, Silicon photonics, Nanophotonics and plasmonics, Photonic devices, Optical sensors

## Abstract

Polarization-resolved control and measurement of optical fields are essential for a wide range of photonic systems, including coherent communication, polarimetric sensing, and quantum information processing. We present a photonic integrated circuit architecture that enables the generation and analysis of arbitrary polarization states. The device provides reconfigurable access to the full polarization degree of freedom of coherent light within a single integrated platform. We experimentally demonstrate arbitrary polarization state generation spanning the Poincaré sphere, as well as Stokes vector measurement on a chip. Unlike conventional Stokes measurements that rely on direct detection, the polarization state in this architecture is inferred from the phase settings required to interferometrically combine polarization-demultiplexed optical fields. As a result, the optical signal itself does not need to be detected or absorbed and remains available for subsequent optical-domain processing. The devices are fabricated in a commercial foundry using CMOS-compatible processes, enabling scalable and reproducible integration. By combining polarization generation and analysis in a compact and stable photonic circuit, this work eliminates the need for external polarization optics and provides a foundation for robust, polarization-enabled photonic integrated systems.

## Abstract (web summary)

A single photonic integrated circuit enables both generation and analysis of arbitrary optical polarization states using a polarization-splitting grating coupler and reconfigurable Mach-Zehnder interferometer mesh, eliminating external polarization optics.

## Graphical abstract

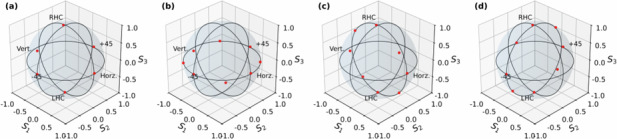

## Introduction

Polarization is a fundamental degree of freedom of light and plays a central role in a wide range of optical systems, from classical and quantum communications^1–3^ to sensing^4^, imaging^5–7^, and metrology^8^. The ability to generate, manipulate, and analyze arbitrary polarization states enables advanced functionalities such as polarization-division multiplexing, polarization-resolved coherent detection, ellipsometry, and polarimetric imaging. As photonic systems continue to scale in complexity and performance, precise and reconfigurable control over polarization has become increasingly important.

An integrated platform that can both generate and analyze arbitrary polarization states enables a self-contained polarimetric system, eliminating the need for external optics and simplifying system-level integration. Such capability is particularly attractive for applications requiring real-time polarization tracking, adaptive compensation, or compact instrumentation, including coherent transceivers, polarization-sensitive sensors, and on-chip quantum photonic circuits. By bringing polarization control and measurement onto a single chip, polarimetric photonic integrated circuits (PICs) open new opportunities for scalable and robust polarization-enabled photonic systems.

Several methods for integrated photonic polarization analyzers and generators have been investigated previously. In particular, metasurface-based analyzers that operate by spatially demultiplexing polarization components have shown great success^6,7,9–11^. However, these devices often act as components in a larger bulk optics system. Fully integrated polarization analyzers on PIC platforms have also been demonstrated^12–16^; however, these devices operate solely with passive, fixed operation components and lack the reprogrammability required to operate as a polarization generator. Polarization-sensitive measurements in beams have also been demonstrated using interferometer meshes fed by sets of grating couplers at different angles. While these systems are capable of performing polarization analysis and generation, the use of single polarization grating couplers makes decoupling polarization and spatially encoded information challenging^17^.

**Fig. 1:**
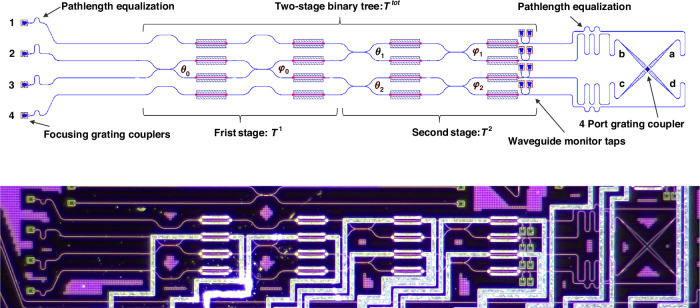
. **Schematic diagram and optical micrograph of the integrated photonic polarization synthesizer/analyzer** The architecture relies on a two-stage binary tree of Mach-Zehnder Interferometers interfaced with the four ports of a normal incidence polarization splitting grating coupler

We have developed a compact integrated photonic architecture based on reprogrammable, bidirectional photonic meshes for generating and analyzing arbitrary polarization states. The architecture relies on two key components: a normally incident polarization splitting grating coupler (PSGC)^15,18,19^ and a photonic mesh of Mach-Zehnder Interferometers (MZIs)^20–23^ arranged in an established topology that supports self-configuration algorithms. The schematic and optical micrograph of this architecture are displayed in Fig. 1. The PSGC we have designed is a symmetric, four-port device that performs two operations. It nominally acts as a polarizing beam splitter, spatially demultiplexing the horizontally and vertically polarized components of an input free-space beam. Secondarily, the device acts as a polarization rotator, coupling both horizontally and vertically polarized light into the quasi-TE mode of their respective waveguide ports. Thus, the PSGC enables the decomposition of any free-space beam into the fundamental quasi-TE mode at each of the device’s four ports. The relatively complex amplitudes that describe each of the device’s four ports then fully characterize the input polarization state. Further, as the device has been designed to couple both horizontally polarized and vertically polarized light at the same input angle (normal incidence), this system enables the independent treatment of spatial and polarization modes.

When operated as an analyzer, we employ the photonic mesh of MZIs as an optical signal processing unit, leveraging self-configuration algorithms^21,24,25^ to recombine the outputs of the PSGC into a single output of the PIC. The phase shifter settings required to interferometrically recombine the outputs of the PSGC into a single output port of the PIC contain the information needed to determine the input polarization state. Unlike typical Stokes parameter measurement techniques, this method does not require the direct detection of the beam being analyzed, such that any input signal is preserved for further optical signal processing by spectroscopy^26,27^, spatial mode analysis^28,29^, or other coherent detection methods^30,31^. The benefit of a self-configuring topology, such as the binary tree photonic mesh used in this work, is that it enables the independent and sequential treatment of each MZI in the system^24,32^. Progressive optimization of the two phase shifters $\phi$ and $\theta$ within each MZI results in the reliable convergence of phase shifter settings, even in the presence of non-ideal components^22,25,33,34^. When operated as a polarization synthesizer, the photonic mesh of MZIs is used to generate arbitrary sets of complex amplitudes at the ports of the PSGC. Calibration of each phase shifter of the photonic mesh generates a mapping between electrical control signals and the applied relative phase shift, resulting in programmatic control of the launched polarization state. In this work, we operate the device in both configurations, demonstrating the efficacy of this architecture for polarization analysis and generation.

While the individual building blocks employed in this work, self-configuring interferometric meshes and polarization splitting grating couplers, have been previously demonstrated, the contribution of this work is architectural and functional. By combining a polarization-degenerate, normal-incidence PSGC with a reconfigurable interferometric mesh, we realize a unified platform capable of both polarization synthesis and analysis within the same device. This bidirectional functionality is not accessible in conventional passive polarimeters or fixed polarization controllers, as it would require precise phase and amplitude control off-chip.

## Results

### Polarization splitting grating coupler

While prior work has established polarimetric architectures based on two-port PSGCs combined with tunable Mach–Zehnder interferometers^35^, the four-port PSGC used in this work is motivated by enabling efficient operation at normal incidence. Conventional two-port PSGCs typically operate at an oblique coupling angle to suppress second-order back reflections. In this configuration, the coupling geometry defines a plane of incidence, and the two orthogonal polarization components couple through diffraction processes occurring in orthogonal planes. As a result, a PSGC optimized for a coupling at an angle in one plane will couple the orthogonal polarization at the same angle but in a rotated plane, preventing a single incident beam or fiber from simultaneously satisfying the optimal coupling condition for both polarization states.

Normal incidence represents a unique configuration in which this limitation disappears as the coupling geometry becomes rotationally degenerate, allowing both orthogonal polarization states to satisfy identical coupling conditions simultaneously. Achieving this polarization-degenerate coupling condition requires the enforcement of horizontal symmetry in the grating structure, which naturally produces a four-port coupling configuration in which opposing waveguides carry equal-amplitude representations of the corresponding polarization components. In addition to improving coupling efficiency relative to oblique-incidence PSGCs, normal-incidence operation is particularly attractive for interfacing with free-space beams, as it simplifies optical alignment and enables straightforward coupling to vertically incident fibers or collimated beams.

**Fig. 2:**
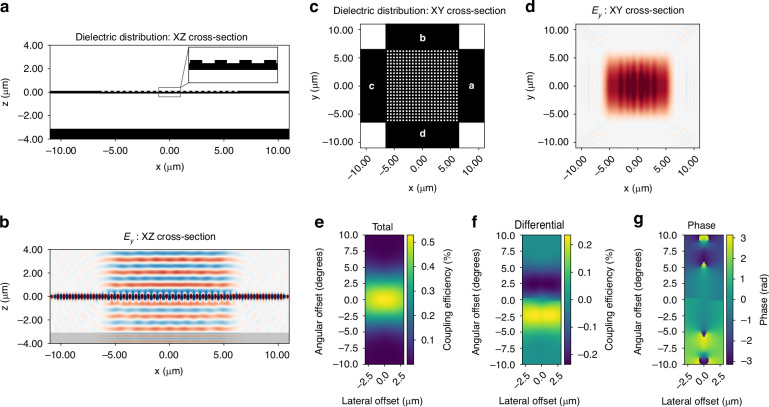
Finited Differenc Time Domain modelling of the polarization splitting grating coupler. **a** X-Z cross-section of the uniform PSGC with a 70 nm partial etch. Inset: Zoom in on several local periods of the PSGC. **b** X-Z Cross-section of the FDTD simulations of the device at 1.550 ${\rm{\mu }}{\rm{m}}$ with a simulated insertion loss of −2.9 dB. Roughly half of the light is lost toward the substrate as a result of the vertical symmetry of the grating layer. **c** X-Y cross-section of the uniform PSGC showing four-fold symmetry. **d** X-Y cross-section of the FDTD simulations displaying the emission profile of the grating when excited from the left and right ports with equal amplitude, in-phase modes. The emitted grating profile has a 86.4% mode overlap with a 10.4 ${\rm{\mu }}{\rm{m}}$ MFD Gaussian mode. **e** Simulated total coupling efficiency across two opposing ports for a horizontally polarized input beam as a function of angular and lateral offset. **f** Differential coupling efficiency between opposing ports for a horizontally polarized input beam as a function of angular and lateral offset. **g** Relative phase between opposing ports for a horizontally polarized input beam as a function of angular and lateral offset

The PSGC used in this work is designed by enforcing four-fold symmetry onto a typical uniform Bragg’s grating coupler. This device leverages the horizontal symmetry of a normal-incidence beam centered on a two-dimensional square lattice grating coupler. This principle is shown in Fig. 2a, b, where a normal-incidence beam, which has a dominant field component that is out of plane (${{\boldsymbol{E}}}_{{\boldsymbol{y}}}$), is centered on a horizontally symmetric grating. It can be observed that the beam is coupled equally into the fundamental quasi-TE mode at the two opposing ports of the device. In accordance with the horizontal symmetry of this system, the two coupled modes will be completely in phase. Likewise, a centered normal-incidence beam that has a dominant field component along the x-axis (${{\boldsymbol{E}}}_{{\boldsymbol{x}}}$) will be evenly split, with zero relative phase, between the quasi-TE modes of the opposing vertical ports due to the ${90}^{\circ }$ rotational symmetry of the system.

The PSGC is designed for a 220 nm thick silicon on insulator (SOI) platform, with a 3 ${\rm{\mu }}{\rm{m}}$ thick buried oxide layer. The grating etch depth is set to 70 nm, as determined by the available processes provided by the Multi-Project Wafer (MPW) shuttle run at Advanced Micro Foundry (AMF) Singapore. We employ slowly varying tapers between the PSGC and SOI single-mode waveguides, which reduce the waveguide width from 13 ${\rm{\mu }}{\rm{m}}$ at the PSGC to 500 nm over a 150 ${\rm{\mu }}{\rm{m}}$ transition^36^. The PSGC waveguide width was chosen to be 13 ${\rm{\mu }}{\rm{m}}$ to closely match the field profile associated with a single-mode fiber at normal incidence. A 50% fill factor was chosen for the gratings so that both the minimum silicon feature size and the minimum etch feature size would be well within the fabrication tolerances provided by the MPW run. The grating period is determined via the grating equation^37^, given in Eq. 1, which imposes phase-matching conditions within each unit cell of the grating. Here, ${n}_{{eff}}$ represents the effective index of the unperturbed waveguide mode, ${n}_{{pert}}$ represents the effective index of the perturbed waveguide mode, ${n}_{{clad}}$ represents the index of the cladding, $D$ is the fill factor, ${\theta }_{{grat}}$ is the coupling angle of the grating, $\lambda$ is the operating wavelength, and $\Lambda$ is the grating period. Solving Eq. 1 given the set of existing parameters at an operating wavelength of 1.55 μm results in a grating period of 556 nm.

1 $${n}_{{eff}}D+{n}_{{pert}}\left(1-D\right)+{n}_{{clad}}\sin \left({\theta }_{{grat}}\right)=\frac{m\lambda }{\Lambda }$$

The coupler is modeled using a 3-dimensional finite-difference time-domain (FDTD) method with Tidy3D^38^. Figure 2c provides a cross-section of the modeled geometry in the silicon device layer. The emission profile of the grating is simulated by launching the quasi-TE mode with equal phase from each of the horizontal ports of the PSGC. Figure 2d displays a cross-section of the simulated field profile 2 ${\rm{\mu }}{\rm{m}}$ above the device layer, within the oxide top cladding. The emission field profile has an 86.4% mode overlap with a 10.4 ${\rm{\mu }}{\rm{m}}$ mode field diameter (MFD) Gaussian field profile, which may be improved in future work by introducing an apodization to the grating design. Furthermore, the coupler exhibits an insertion loss of −2.9 dB (51%) at 1.55 ${\rm{\mu }}{\rm{m}}$ and a minimum insertion loss of −2.6 dB (55%) at 1.535 ${\rm{\mu }}{\rm{m}}$. The efficiency is predominantly limited by large substrate losses, as can be observed in Fig. 2b that result from the nearly perfect vertical symmetry of the device, which is only mildly broken by reflections from the silicon substrate and the use of a 70 nm partial etch in the grating layer. In future work, a number of techniques may be employed to break the vertical symmetry and improve directionality at normal incidence, including metallic bottom reflectors, Bragg bottom reflectors, polysilicon overlays, dual-layer grating, and multi-etch blazed grating structures^18,19,37,39–41^. Application of these techniques has the potential to achieve sub-decibel insertion loss at normal incidence, further enabling the use of this architecture in loss-limited applications such as quantum photonics.

According to the coordinate system we have defined, the complex amplitudes measured through ports **a** & **c**, as shown in Fig. 2c, of the PSGC will characterize any horizontally polarized component of the input beam. Similarly, the complex amplitudes measured through the ports **b** & **d**, as shown in Fig. 2c, of the PSGC will characterize any vertically polarized component of the input beam. The four ports of the PSGC, represented as a four-element vector of complex amplitudes, fully characterize the polarization of a coupled coherent beam. By analyzing these complex amplitudes, one may determine the polarization state of an input beam; conversely, by setting these complex amplitudes, one may synthesize the polarization state of an output beam. Table 1 provides the set of complex amplitudes for each port of the PSGC associated with the fundamental polarization basis states.

**Table 1:** Complex amplitudes of PSGC ports

Pol. State	Port A	Port B	Port C	Port D
Horizontal linear	$\frac{1}{\sqrt{2}}{e}^{j\theta }$	0	$\frac{1}{\sqrt{2}}{e}^{j\theta }$	0
Vertical linear	0	$\frac{1}{\sqrt{2}}{e}^{j\theta }$	0	$\frac{1}{\sqrt{2}}{e}^{j\theta }$
+45 Linear	$\frac{1}{2}{e}^{j\theta }$	$\frac{1}{2}{e}^{j\theta }$	$\frac{1}{2}{e}^{j\theta }$	$\frac{1}{2}{e}^{j\theta }$
−45 Linear	$\frac{1}{2}{e}^{j\theta }$	$\frac{1}{2}{e}^{j(\theta -\pi )}$	$\frac{1}{2}{e}^{j\theta }$	$\frac{1}{2}{e}^{j(\theta -\pi )}$
Right-hand circular	$\frac{1}{2}{e}^{j\theta }$	$\frac{1}{2}{e}^{j(\theta -\frac{\pi }{2})}$	$\frac{1}{2}{e}^{j\theta }$	$\frac{1}{2}{e}^{j(\theta -\frac{\pi }{2})}$
Left-hand circular	$\frac{1}{2}{e}^{j\theta }$	$\frac{1}{2}{e}^{j(\theta +\frac{\pi }{2})}$	$\frac{1}{2}{e}^{j\theta }$	$\frac{1}{2}{e}^{j(\theta +\frac{\pi }{2})}$

If the central-symmetric condition of the system is broken by introducing a lateral or angular offset to the beam under analysis, we observe three dominant effects on the performance of the PSGC. First, the total insertion loss, split between each of the ports of the PSGC, increases as the beam trends away from being centered and normal incidence, as can be seen in Fig. 2e. This effect is significantly more sensitive to angular misalignment than lateral misalignment, with the insertion loss increasing to −10 dB (10%) for a $\pm {5}^{\circ }$ misalignment, while increasing to −3.5 dB (44%) for a ±3 ${\rm{\mu }}{\rm{m}}$ misalignment. Second, as the angle of incidence increases in a particular plane, so does the preferential illumination between the ports corresponding to that plane. This trend is bound by the overall decrease in coupling efficiency at large angles of incidence. Figure 2f displays this effect by plotting the difference in coupling efficiency between ports **a** & **c** for a horizontally polarized input. Lastly, we observe the introduction of a relative phase shift between opposing ports of the PSGC as a function of lateral and angular offset. From Fig. 2g we note that the introduced phase shift is relatively small over the angular and lateral offset range for which there is non-negligible coupling efficiency. For the purposes of analytically modeling this PIC architecture, we will assume that a central-symmetric condition is enforced in the nominal case and discuss the implications of angular or lateral offsets toward polarization accuracy.

### Binary tree photonic mesh

The challenge of measuring and generating complex vectors is well addressed by photonic meshes, which are networks of MZIs arranged in particular architectures^20,21^. Within a photonic mesh, each MZI is comprised of a nominal 50:50 beam splitter, an internal tunable phase shifter $\theta$, a nominal 50:50 beam combiner, and an external tunable phase shifter $\phi$. By adjusting the $\theta$ phase shifter of an MZI, one may arbitrarily adjust the splitting ratio of an input to the MZI between its two output ports. By adjusting the $\phi$ phase shifter, one may arbitrarily adjust the relative phase between the two output ports of an MZI. Equation 2 gives the transmission matrix of such an MZI, demonstrating that by adjusting the values of $\theta$ and $\phi$ accordingly, any linear, unitary, $2\times 2$ matrix transformation may be generated. In Eq. 2, *B* represents the transfer matrix of an ideal 50:50 beam splitter/combiner, $T(\theta )$ and $T(\phi )$ represent the transfer matrices of the internal and external phase shifters, respectively, and *i* is an indexing variable.

2 $$\begin{array}{c}T\left(i\right)={T}_{\phi }B{T}_{\theta }B\\ =\frac{1}{2}\left[\begin{array}{cc}{e}^{j{\phi }_{i}} & 0\\ 0 & 1\end{array}\right]\left[\begin{array}{cc}1 & j\\ j & 1\end{array}\right]\left[\begin{array}{cc}{e}^{j{\theta }_{i}} & 0\\ 0 & 1\end{array}\right]\left[\begin{array}{cc}1 & j\\ j & 1\end{array}\right]\\ =j{e}^{\frac{j{\theta }_{i}}{2}}\left[\begin{array}{cc}{e}^{j{\phi }_{i}}\sin (\frac{{\theta }_{i}}{2}) & {e}^{j{\phi }_{i}}\cos (\frac{{\theta }_{i}}{2})\\ \cos (\frac{{\theta }_{i}}{2}) & -\sin (\frac{{\theta }_{i}}{2})\end{array}\right]\end{array}$$

Photonic meshes enable the extension of these linear, unitary operators from $2\times 2$ matrix transformations to arbitrarily large *N×N* transformations^33^. Here, we employ a two-stage binary tree^20,23,24^, depicted in Fig. 1, to operate on the four-element vector that represents the ports of the PSGC. The use of a two-stage binary tree mesh is motivated by the four-port nature of the PSGC, which produces two pairs of outputs corresponding to orthogonal polarization components. This architecture represents the minimal reconfigurable network that supports the self-configuration algorithms required to coherently combine and control all four channels, enabling full access to the polarization state space. The transfer matrix of a binary tree with multiple stages may be represented by the product of matrices that represent each intermediate stage of the mesh. Equation 3 gives the transfer matrix of a two-stage binary tree when operated from left to right, as displayed in Fig. 1 where ${T}^{1}$, ${T}^{2}$, and ${T}^{{tot}}$ represent the transfer matrix operations of the first stage of the binary tree mesh, second stage of the binary tree mesh, and the total operation of the two-stage binary tree respectively, and the terms ${T}_{{xy}}(i)$ represent the individual matrix elements of a single MZI with corresponding phase shifters ${\theta }_{i}$ and ${\phi }_{i}$.

3 $$\begin{array}{c}{T}^{{Tot}}={T}^{2}{T}^{1}\\ =\left[\begin{array}{cccc}{T}_{11}(1) & {T}_{12}(1) & 0 & 0\\ {T}_{21}(1) & {T}_{22}(1) & 0 & 0\\ 0 & 0 & {T}_{11}(2) & {T}_{12}(2)\\ 0 & 0 & {T}_{21}(2) & {T}_{22}(2)\end{array}\right]\left[\begin{array}{cccc}1 & 0 & 0 & 0\\ 0 & {T}_{11}(0) & {T}_{12}(0) & 0\\ 0 & {T}_{21}(0) & {T}_{22}(0) & 0\\ 0 & 0 & 0 & 1\end{array}\right]\\ =\left[\begin{array}{cccc}{T}_{11}(1) & {T}_{12}(1){T}_{11}(0) & {T}_{12}(1){T}_{12}(0) & 0\\ {T}_{21}(1) & {T}_{22}(1){T}_{11}(0) & {T}_{22}(1){T}_{12}(0) & 0\\ 0 & {T}_{11}(2){T}_{21}(0) & {T}_{11}(2){T}_{22}(0) & {T}_{12}(2)\\ 0 & {T}_{21}(2){T}_{21}(0) & {T}_{21}(2){T}_{22}(0) & {T}_{22}(2)\end{array}\right]\end{array}$$

From Eq. 3 as well as Fig. 1, it can be determined that this architecture of photonic mesh enables an input beam incident on either focusing grating couplers 2 or 3 to be arbitrarily distributed over the ports of the PSGC and vice versa. For the purposes of this work, we will always treat focusing grating coupler 2 as the input/output port of the PIC, although this choice is ultimately arbitrary. Under these conditions, the vector of complex amplitudes that represents the ports of the PSGC can be expressed as functions of ${\theta }_{i}$ and ${\phi }_{i}$.

4 $$\left[\begin{array}{c}a\\ b\\ c\\ d\end{array}\right]=\left[\begin{array}{c}-{e}^{j\left({\phi }_{0}+{\phi }_{1}\right)}{e}^{\frac{j}{2}\left({\theta }_{0}+{\theta }_{1}\right)}\sin (\frac{{\theta }_{0}}{2})\cos (\frac{{\theta }_{1}}{2})\\ {e}^{j{\phi }_{0}}{e}^{\frac{j}{2}\left({\theta }_{0}+{\theta }_{1}\right)}\sin (\frac{{\theta }_{0}}{2})\sin (\frac{{\theta }_{1}}{2})\\ {-e}^{j{\phi }_{2}}{e}^{\frac{j}{2}\left({\theta }_{0}+{\theta }_{2}\right)}\cos (\frac{{\theta }_{0}}{2})\sin (\frac{{\theta }_{2}}{2})\\ {-e}^{\frac{j}{2}\left({\theta }_{0}+{\theta }_{2}\right)}\cos (\frac{{\theta }_{0}}{2})\cos (\frac{{\theta }_{2}}{2})\end{array}\right]$$

By controlling the values of ${\theta }_{i}$, ${\phi }_{i}$ over a range of [0,$2\pi$], each element of the complex vector given by Eq. 4 may traverse the perimeter of the unit circle in the complex plane. To determine the appropriate values of ${\theta }_{i}$, ${\phi }_{i}$, we must consider the constraints of the system.

When operating as a symmetric coupler with a centered beam at normal incidence, opposing ports of the PSGC will have equal amplitudes and will be in-phase. To satisfy this condition, it is clear from inspection that the first stage of the binary tree should be biased to the quadrature point of the transmission curve such that ${\theta }_{0}=\frac{\pi }{2}\pm 2\pi n$. The values of ${\theta }_{1}$ and ${\theta }_{2}$ are then determined by the ratio of vertically polarized light to horizontally polarized light. In accordance with the coordinate system that we have defined, wherein ports **a** and **c** are associated with horizontally polarized light and ports **b** and **d** are associated with vertically polarized light, the phase shifter values ${\theta }_{1}$ and ${\theta }_{2}$ are given by Eqs. 5 and 6 respectively.

5 $${\theta }_{1}=2\,{\cos }^{-1}(\sqrt{\frac{{I}_{=}}{{I}_{=}+{I}_{{\rm{||}}}}})\pm 2\pi n$$

6 $${\theta }_{2}=2\,{\sin }^{-1}(\sqrt{\frac{{I}_{=}}{{I}_{=}+{I}_{{\rm{||}}}}})\pm 2\pi n$$

Here I_=_ denotes the intensity of horizontally polarized light and ${I}_{{||}}$ denotes the intensity of vertically polarized light. The fraction of horizontally polarized light can be varied continuously between [0, 1] as the values of ${{\rm{\theta }}}_{1}$ and ${{\rm{\theta }}}_{2}$ are varied simultaneously between $[0,{\rm{\pi }}]$ and $[{\rm{\pi }},0]$ respectively.

In order to maintain constructive interference of the vertically polarized components between ports **b** and **d**, the value of ${{\rm{\phi }}}_{0}$ must be set to compensate for the phase difference introduced by the settings of ${{\rm{\theta }}}_{1}$ and ${{\rm{\theta }}}_{2}$. By taking the difference in the angular arguments of components **b** and **d** in Eq. 4, the following phase matching condition may be defined.

7 $${\phi }_{0}=\frac{{\theta }_{2}}{2}-\frac{{\theta }_{1}}{2}\pm \left(2n\pm 1\right)\pi$$

Similarly, the settings of phase shifters ${{\rm{\phi }}}_{1}$ and ${{\rm{\phi }}}_{2}$ must be adjusted to maintain constructive interference of the horizontally polarized components between ports **a** and **c**. By taking the difference in the angular arguments of components **a** and **c** in Eq. 4, the analogous phase matching condition may be defined.

8 $${\phi }_{1}-{\phi }_{2}=\frac{{\theta }_{2}}{2}-\frac{{\theta }_{1}}{2}-{\phi }_{0}$$

Under the assumption that the phase matching condition described in Eq. 7 is satisfied, this may be simplified further to:

9 $${\phi }_{1}-{\phi }_{2}=\pm \left(2n\pm 1\right)\pi$$

Finally, from Eq. 4, the relative phase between horizontally and vertically polarized light can be expressed as either ${\phi }_{1}$ or ${\phi }_{2}$. By adjusting both terms simultaneously, by the same amount and in the same direction, any relative phase between the orthogonal polarization basis may be synthesized. According to these principles, by adjusting the phase shifter settings of the binary tree, any arbitrary polarization state may be mapped between the ports of the PSGC and a single input channel of the PIC.

Thus far, this model has made two simplifying assumptions: that each beam splitter in the photonic mesh is an ideal 50:50 coupler and that a central-symmetric condition has been enforced. Violation of either of these assumptions introduces variation from the idealized model discussed above. In the case of non-ideal beam splitters, the achievable extinction ratio of each MZI within the photonic mesh will be reduced^25,33,34^. Ultimately, this will limit the ability of the photonic mesh to generate or analyze purely horizontal or purely vertical polarization states while having a negligible impact on any intermediate linear polarization states, circular polarized light or arbitrary elliptical polarization states.

As discussed in the analysis of the PSGC, the introduction of a lateral or angular offset results in a reduction in the total coupling efficiency, preferential illumination of a subset of opposing ports, and the introduction of a relative phase shift between opposing ports. The latter two of these effects solely impact the relatively complex amplitude of opposing ports, whereas polarimetric operations in this architecture are governed by the relative complex amplitudes of orthogonal ports. As such, any imbalanced amplitude or phase between opposing ports of the PSGC can be compensated for via the programmable phase shifters of the photonic mesh without impacting operation.

The dominant impact of violating the central symmetric condition is a potential imbalance of the relative coupling efficiency between orthogonal ports caused by independent offsets in the XZ-plane and the YZ-plane. Should the independent offsets in the XZ-plane and YZ-plane be equal in magnitude, regardless of polarity, this effect will impact each set of orthogonal ports equally, resulting in a negligible impact on polarimetric operations. However, in the case that these independent offsets are not equal in magnitude, the differential insertion loss between orthogonal ports would present itself as an error in the approximated ratio of horizontally to vertically polarized light.

### Polarimetric operation

To experimentally validate the analytical principles described above, we fabricated a proof-of-concept device through a commercial foundry (AMF, Singapore). For this demonstration, we employ a 220 nm SOI platform with thermo-optic phase shifters, owing to its maturity, reliability, and commercial accessibility. This choice provides a robust and well-understood testbed for demonstrating the proposed functionality, although it introduces known trade-offs in propagation loss and actuation speed that are not intrinsic to the architecture and will be discussed in detail in the following sections.

To begin operation of the PIC architecture, each phase shifter in the photonic mesh is calibrated to generate a mapping between the applied heater power and the generated relative phase shifts $\theta$ and $\phi$ (detailed in the “Materials and methods” section). This calibration procedure allows for automated compensation of the initial phase imbalances within each balanced MZI, which may result from fabrication variations between the two arms of the MZI. The combination of this mapping between electrical input and optical phase with the derived analytical model allows for the generation and analysis of arbitrary polarization states.

**Fig. 3:**
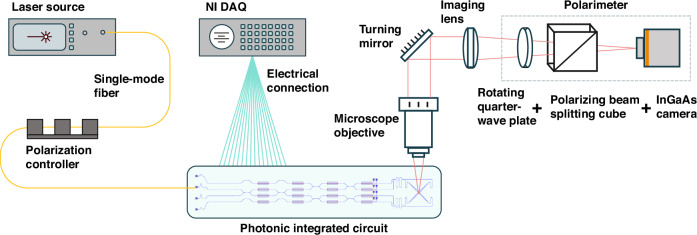
. **Experimental setup for characterizing the PIC as an arbitrary polarization synthesizer** The fundamental quasi-TE mode of the PIC is excited via a fiber-coupled external laser source. The phase shifter settings of the photonic mesh are managed via a National Instruments DAQ, which provides full control over the complex amplitudes entering the four ports of the PSGC. The output of the PSGC is imaged onto a polarimeter, which operates on the principles of the rotating quarter-wave plate method

The principles of the arbitrary polarization generator have been tested using the experimental setup detailed in Fig. 3. We characterize the output polarization state of the PIC by imaging the emissions of the PSGC onto a rotating quarter-wave plate polarimeter^42^. For each measurement, the quarter-wave plate is rotated between ${0}^{\circ }$ and ${180}^{\circ }$ in ${10}^{\circ }$ increments while recording the power transmitted through a polarizing beam splitting cube, which acts as a linear polarizer. The relative intensity is recorded using a Bobcat 640 InGaAs camera by integrating over the pixels associated with the output of the PSGC. Each rotation of the quarter-wave plate is repeated over 10 samples for averaging. From the recorded intensity as a function of the quarter-wave plate angle, one can extract the Stokes Parameters for the polarization state under analysis.

**Fig. 4:**
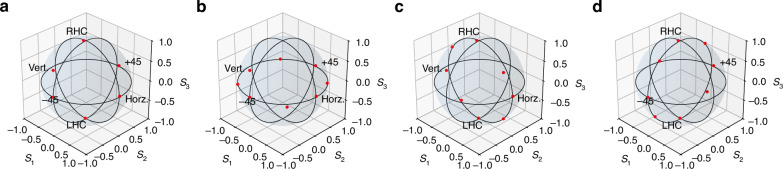
. **Generated states as characterized by the rotating quarter-wave plate polarimeter** **a** Sets of basis pairs demonstrating horizontal and vertical linear polarization states, positive and negative 45° linear polarization states, as well as right-hand circular and left-hand circular polarization states. **b** Intermediate linear polarization states between the horizontal, vertical, positive and negative ${45}^{\circ }$ linear poles. **c** Intermediate elliptical polarization states between the right-hand circular, left-hand circular, positive and negative ${45}^{\circ }$ linear poles. **d** Intermediate elliptical polarization states between the horizontal, vertical, right- and left-hand circular poles

Using this technique, we verify the six fundamental polarization basis states described in Table 1. We have plotted the normalized Stokes Parameters on the Poincaré sphere in Fig. 4a. It can be seen that the device is capable of generating each of the common polarization basis pairs: horizontal and vertical linear polarizations, positive and negative ${45}^{\circ }$ linear polarizations, and left- and right-hand circular polarizations. The accuracy of the polarization generator is evaluated using the root-mean-square error (RMSE) between the measured normalized Stokes vector and the expected value, which we measure to be 0.059. This metric corresponds to the geometric distance between points on the Poincaré sphere and, for small deviations, can be interpreted as an angular error. It is anticipated that this residual may be reduced in future work through an additional layer of calibration, thereby generating a direct mapping between the applied electrical signals and the generated polarization state, rather than relying on an idealized analytical model.

By appropriately tuning the binary tree mesh, we can generate the superposition of any subset of these basis functions, thus generating any polarization state on the Poincaré sphere. We demonstrate this by synthesizing intermediate polarization states between subsets of each of the basis pairs. In Fig. 4b, we generate states between the horizontal linear, positive ${45}^{\circ }$ linear, vertical linear, and negative ${45}^{\circ }$ linear polarization states. In Fig. 4c, we generate states between the positive ${45}^{\circ }$ linear, right-hand circular, negative ${45}^{\circ }$ linear, and left-hand circular polarization states. And in Fig. 4d, we generate states between the horizontal linear, right-hand circular, vertical linear, and left-hand circular polarization states.

To demonstrate the polarization analysis capabilities of the PIC, the system is operated in the reverse direction, from right to left, as depicted in Fig. 1. Light is coupled into the PSGC via a cleaved single-mode fiber aligned at normal incidence and positioned using a Thorlabs Nanomax 600 series 6-axis stage. Light coupled to the four ports of the PSGC is analyzed using a self-configuration algorithm^24^ based on the power minimization of light out of focusing grating couplers 1, 3, and 4. This algorithm sequentially optimizes the phase shifter settings at ${\theta }_{1},{\phi }_{1}$ to minimize power at port 1, ${\theta }_{2},{\phi }_{2}$ to minimize power at port 4, and ${\theta }_{0},{\phi }_{0}$ to minimize power at port 3. The relative intensity at each of these ports is monitored simultaneously by imaging each of the focusing grating couplers onto the InGaAs camera. This process enables us to direct all power from the ports of the PSGC to focusing grating coupler 2 without the need for direct detection of the signal under analysis. The phase shifter settings required to map all power from the four ports of the PSGC to focusing grating coupler 2 provide the necessary information to reconstruct the complex amplitudes at the PSGC via Eq. 4. This eliminates the need for direct detection of the beam output from focusing grating coupler 2, preserving it for further optical domain signal processing.

**Fig. 5:**
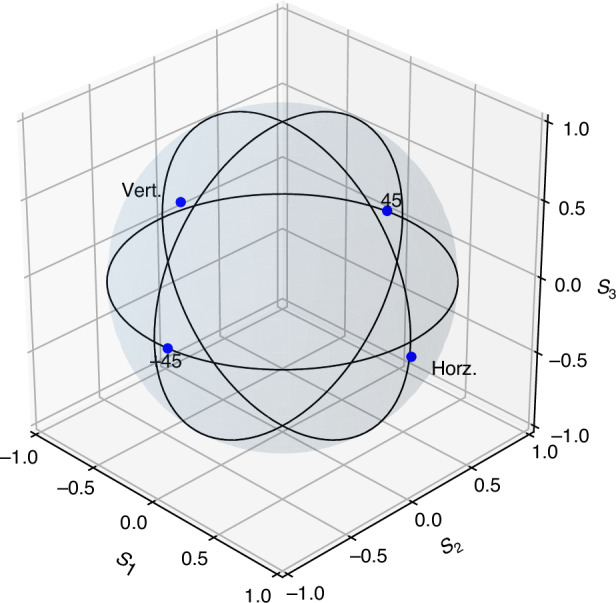
. **Normalized Stokes parameters measured by operating the PIC as a polarization analyzer** The input polarization states are set to horizontal, vertical, positive and negative ${45}^{\circ }$ linear by aligning the input fiber to test structures designed to accept linear polarization states

To launch known polarization states into the PSGC, we use test structures comprising single-polarization grating couplers designed with known rotation angles relative to the PSGC. While the cleaved single-mode fiber is aligned to such a test structure, a manual polarization controller is adjusted to maximize coupling through the single-polarization grating. This enables the accurate alignment of a given polarization state before analysis via the PIC. Figure 5 displays the normalized Stokes parameters extracted via this technique for the horizontal, vertical, positive ${45}^{\circ }$, and negative ${45}^{\circ }$ linear polarizations. The figure of merit for the polarization analyzer is defined similarly to that of the polarization generator. The RMSE between the analyzed Stokes vector and the expected value is 0.121. Again, it is anticipated that these errors may be reduced in future work by calibrating the devices against a set of known polarization states.

Throughout this demonstration, the self-configuration procedure employed in polarization analysis was observed to converge reliably in a single pass through the tuning sequence, without requiring iterative refinement. We did not observe sensitivity to the initial phase settings or evidence of sensitivity to other minima, which we attribute in part to the use of thermal isolation trenches that reduce crosstalk between phase shifters. The total time required for a single polarization analysis measurement is currently on the order of 1 min. We emphasize that this is not limited by the intrinsic response time of the thermo-optic phase shifters $(\approx 30{\rm{\mu }}{\rm{s}}$), but rather by the latency of the NI DAQ used for control electronics and measurement interface. Faster operation is therefore achievable with optimized control hardware, as demonstrated in prior work using FPGA-based implementations for programmable photonic meshes^23,43^. Further, these algorithms may be continuously run for a form of closed-loop operation, as has been demonstrated on similar architectures^23,43^, enabling continuous polarization tracking, conversion, and compensation.

**Fig. 6:**
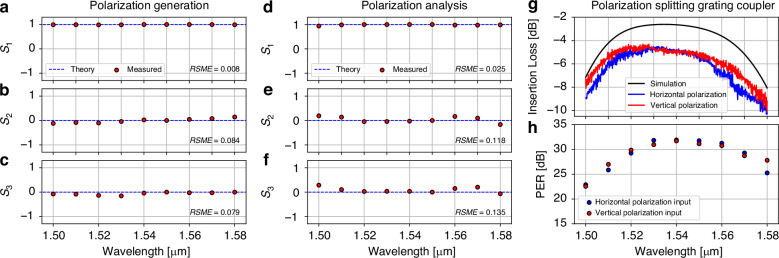
. **Wavelength dependence of the polarization generator** **a**–**c** and the polarization analyzer. **d**–**f** We observe small drifts in the normalized Stokes parameters across the operational wavelength band, which is dominated by the ${S}_{2}$ and ${S}_{3}$ parameters. **g** Measured coupling efficiency of the normal incidence PSGC when coupled to a single-mode fiber for the horizontal and vertical polarization states. We observe a minimum insertion loss of −4.5 dB and −4.3 dB for the TE and TM modes, respectively. **h** Polarization extinction ratio of the system, exceeding 20 dB across the wavelength band

In both the polarization generation and analysis cases, the phase shifters elements have been calibrated at an operating wavelength of 1.55 ${\rm{\mu }}{\rm{m}}$. To investigate the implicit wavelength dependence of the architecture, we repeat the generation and analysis procedures across a wavelength band spanning 1.50 ${\rm{\mu }}{\rm{m}}$ to 1.58 ${\rm{\mu }}{\rm{m}}$ for a fixed input/output polarization state. We have plotted the variation of each of the normalized Stokes parameters in Fig. 6a–c for the polarization generator and Fig. 6d–f for the polarization analyzer. While the total variation across the band is small, it is noted that the variation in both cases is dominated by the ${S}_{2}$ and ${S}_{3}$ parameters. This is an indication that wavelength-dependent phase error dominates over any wavelength-dependent splitting errors.

To characterize the polarization extinction ratio (PER) of the system, we have launched horizontally polarized light into the PSGC while the photonic mesh is first configured to route horizontally polarized light to the focusing grating coupler 2. By orthogonality, no vertically polarized light shall be routed to this same output. We then reconfigure the mesh to route any vertically polarized light to the focusing grating coupler 2. Similarly, by orthogonality, no horizontally polarized light shall be routed to this same output. The polarization extinction ratio is taken as the ratio of the recorded power in these two configurations. This experiment is then repeated for a vertically polarized input. We observe similar performance for both input polarization states, exhibiting a peak PER of 32dB near the center of the band, with performance decreasing to 22dB near the band edges.

To analyze the insertion loss of the PSGC, we once again operate the system in a forward orientation, from left to right as depicted in Fig. 1. Light is coupled into focusing grating coupler 2, while the binary tree is programmed to transmit horizontally polarized light from the PSGC. We record the transmission as a function of wavelength and extract the coupling efficiency by accounting for the input power spectrum of the tunable laser source and the transmission spectrum of the focusing grating couplers. This process is then repeated while the binary tree is reprogrammed to transmit vertically polarized light from the PSGC. The resulting insertion loss measurements are plotted in Fig. 6g. The PSGC exhibits a similar minimum insertion loss for both polarization states, measuring −4.5 dB and −4.3 dB for the TE and TM modes, respectively. These results represent a notable improvement in coupling efficiency compared to the alternative two-port PSGCs^35^. Additionally, the PSGC maintains a similar 1 dB bandwidth for both polarization states, measuring 42 nm and 45 nm for the TE and TM modes, respectively. However, it can be observed that there is a polarization-dependent loss (PDL) that increases toward the edges of the operational band, with a maximum PDL of 1.2 dB at 1.565 ${\rm{\mu }}{\rm{m}}$. It is possible that the deviation from the expected symmetric coupling of the TE and TM modes is a result of asymmetries introduced in the fabrication process. The impact of the inherent PDL toward the edges of the operational band of the PSGC is similar to that of an offset between the input angles in the XZ and YZ planes. This PDL will be represented as an error in the approximated ratio of horizontally to vertically polarized light, ultimately skewing the results of a polarization state measurement of generation. However, unlike the case of an angular offset, the inherent PDL of the PSGC may be characterized as shown in Fig. 6h and compensated for by adjusting our interpretation of ${\theta }_{1}$ and ${\theta }_{2}$ in Eqs. 5 and 6 respectively, to account for an error term $\Delta {I}_{=}$.

## Discussion

Existing approaches to on-chip polarization control and analysis based on metasurface and passive devices offer compact footprints and low power consumption, but are typically fixed at fabrication and, therefore, lack reconfigurability. We have demonstrated an interferometric mesh-based system that enables dynamic and programmable control over polarization states at the expense of increased complexity and power consumption associated with active phase tuning. Within this architecture, a polarization splitting grating coupler generates a mapping between arbitrary polarization states and a vector of complex amplitudes to be operated on by a binary tree of MZIs. Analysis of the circuit details the underlying principle by which a reconfigurable photonic mesh, coupled to the ports of a four-port PSGC, may analyze and synthesize arbitrary polarization states. Based on these principles, we demonstrate the architecture’s ability to offer flexible and reconfigurable access to the polarization degree of freedom within a compact integrated platform.

When operated as a polarization synthesizer, the phase shifters within the binary tree control both the relative amplitudes and phases of the horizontally and vertically polarized components coupled by the PSGC, allowing the generation of arbitrary polarization states. Conversely, when operated in the reverse direction as a polarization analyzer, the appropriate phase shifter settings are applied to interferometrically route the optical fields from each PSGC port to a single focusing grating coupler on the PIC. In this configuration, the polarization state incident on the PSGC is fully characterized by the phase shifter settings required to achieve constructive interference at the output. Because the polarization information is encoded in these settings rather than in a directly detected optical signal, the architecture naturally enables further downstream optical processing, in essence acting as a convenient coupling interface for any integrated photonic circuit while simultaneously performing the operations of a polarization analyzer.

We experimentally demonstrate the device's performance by synthesizing each of the fundamental polarization basis states, including horizontal and vertical linear polarization, ±45° linear polarization, and left- and right-hand circular polarization. By characterizing the output polarization states, we determine that the device can accurately control the relative amplitude and phase between horizontal and vertical polarizations, with an RMSE of 0.059 across Stokes vectors. We further demonstrate the generation of arbitrary polarization states through controlled superposition of these basis states, spanning the full Poincaré sphere. By exploiting the circuit's bidirectional operation, we also use the device as a polarization analyzer and experimentally measure Stokes parameters for a limited set of input polarization states. In the present implementation, known input polarization states were generated by coupling light into a single polarization grating coupler, with the coupler's rotation controlled relative to the PSGC under test. This approach provides a convenient and stable method for generating well-defined linear polarization states, but does not readily enable the generation of arbitrary elliptical or circular states without significant modification of the experimental setup. Despite this limitation in input state preparation, the underlying architecture is based on a linear, reciprocal optical transformation. As a result, the capability to synthesize arbitrary polarization states, as demonstrated experimentally across the full Poincaré sphere, implies the ability to analyze arbitrary input polarization states using the same calibrated transformation. For the set of polarization states we have analyzed, we estimate an RMSE of 0.121.

In the context of coherent communication systems, such deviations would lead to residual crosstalk between polarization channels, while in polarimetric sensing applications, they directly reflect reconstruction accuracy. The reported Stokes vector RMSE corresponds to polarization state errors on the order of a few degrees on the Poincaré sphere. In coherent communication systems, such errors may be compensated by digital signal processing and would result in only modest performance penalties. For polarimetric sensing, this level of accuracy is consistent with other integrated photonic implementations^12–14^, although it remains above the precision of bulk optical ellipsometry systems, which typically require sub-degree accuracy^44^. The larger error observed in analyzer operation compared to synthesis is attributed in part to the degree to which we are able to experimentally guarantee the central symmetric condition, as well as the increased sensitivity of the self-configuration and reconstruction process to measurement noise and calibration imperfections. We expect that in future work, the accuracy of these devices may be improved through additional calibration efforts to generate direct mappings between the applied electrical signals and polarization states, as opposed to relying on an idealized analytical model.

The present implementation is realized on a SOI platform utilizing single-mode channel waveguides and thermo-optic phase shifters, resulting in waveguide propagation losses on the order of ~2 dB/cm and actuator power consumption and response times of 18 mW and 30 ${\rm{\mu }}{\rm{s}}$ respectively^26^. While these characteristics are typical for the platform and sufficient for a proof-of-principle demonstration, they do not represent fundamental limitations of the proposed architecture. The underlying approach is fully compatible with alternative photonic platforms and tuning mechanisms. In particular, low-loss platforms such as silicon nitride can significantly reduce propagation loss, enabling improved overall system efficiency and scalability. Similarly, the interferometric mesh can be actuated using faster and more energy-efficient mechanisms, including carrier-based (plasma dispersion) phase shifters, electro-optic materials, or micro-electromechanical systems (MEMS), depending on the requirements of a given application. However, in many relevant applications, including polarization control in coherent communication receivers, polarimetric sensing systems, and state preparation in photonic quantum experiments, polarization compensation typically occurs on timescales associated with environmental drift (milliseconds to seconds), for which thermo-optic tuning is more than sufficient. These considerations highlight that the performance of the demonstrated system can be substantially enhanced through platform and device-level optimization without altering the core architectural principles.

Within this implementation, we measure minimum insertion losses of −4.3 dB and −4.5 dB for the TE and TM polarization components, respectively. The discrepancy between the measured insertion loss of the PSGC and the simulated value of −2.9 dB can be attributed to fabrication tolerances in the grating dimensions, etch depth, and sidewall angles, as well as non-ideal mode matching; factors that are not captured in the idealized simulation model. Despite these non-idealities, the measured insertion loss is an improvement over that of the typical focusing grating coupler that we employ, with the added benefit of coupling at normal incidence. However, in its current state, the passive insertion loss of this PSGC design, alongside that of the focusing grating couplers and waveguide propagation loss, would be limiting for insertion loss-sensitive applications such as coherent communications, astro-photonics, and quantum photonics. In future implementations, the insertion loss of the PSGC and focusing grating coupler may be reduced dramatically through the use of multiple etch steps or additional material layers to break the vertical symmetry. These methods may also serve to improve the accuracy of the device regarding polarization generation and analysis by reducing the impact of secondary reflections and scattering from the substrate.

In conclusion, we have realized a photonic integrated circuit that combines arbitrary polarization generation and polarization analysis within a single integrated platform. By eliminating the need for external polarization optics, this approach reduces system complexity and enables scalable integration with other photonic components fabricated using CMOS-compatible processes. Integrated control and analysis of arbitrary polarization states are particularly relevant for applications that exploit polarization as an information-bearing degree of freedom, including coherent optical communication, polarimetric sensing, and quantum photonic systems. In future work, the scope of polarization analysis technique may be broadened to address partially coherent light by leveraging recently developed techniques for analyzing spatial and temporal coherency^27,30^. The demonstrated platform provides a foundation for compact, self-calibrated polarimetric photonic circuits capable of real-time polarization tracking and adaptive operation, and highlights the growing role of polarization-enabled architectures in advanced integrated photonic systems.

## Materials and methods

Our photonic integrated circuits have been fabricated via the commercial provider Advanced Micro Foundry using a multi-project wafer shuttle run. The devices are fabricated on a 220 nm silicon on insulator wafer using the standard available process steps. An HP 81680 A tunable laser, set to operate at 1.55 ${\rm{\mu }}{\rm{m}}$ and coupled to a single-mode fiber, is used as an external source. We use a Thorlabs FPC562 manual polarization controller to adjust the input polarization state. The fiber output of the manual polarization controller has been cleaved and is aligned to the PIC using a Thorlabs Nanomax 600 series 6-axis stage.

The focusing grating couplers used to interface between single-mode fibers and silicon waveguides on the PIC are designed to emit and accept light at close to 12° from normal incidence at 1.55 ${\rm{\mu }}{\rm{m}}$^23^. These input/output couplers have been characterized to exhibit a minimum insertion loss of −6 dB per coupler at 1.55 ${\rm{\mu }}{\rm{m}}$. The silicon single-mode waveguides used to route light throughout the PIC have a 500 nm $\times$ 220 nm cross-section, which is maintained throughout the photonic mesh.

Every MZI in the two-stage binary tree employs directional couplers as the nominal 50:50 beam splitter and combiner, each with a 300 nm coupling gap and a 40 ${\rm{\mu }}{\rm{m}}$ interaction length. Here we use large radius of curvature bends, R = 35 ${\rm{\mu }}{\rm{m}}$, throughout the circuit to minimize bending losses. The $\theta$ and $\phi$ phase shifters are implemented as titanium nitride heaters, which rely on the thermo-optic effect to adjust the local refractive index of their respective silicon waveguides. Thermal isolation trenches have been etched through the oxide cladding, silicon device layer, buried oxide, and into the silicon substrate on either side of every thermal phase shifter in an attempt to reduce thermal crosstalk between MZIs. We have placed dummy thermo-optic phase shifters, which are not operational, throughout the mesh to loss match every path through the PIC. Additionally, path-length equalization bends have been introduced throughout the mesh to ensure nominal wavelength independence, propagation loss matching, and coherence retention throughout the PIC. Using three dimensional FDTD simulations we predict an insertion loss per MZI to be on the order of 0.05 dB, however in practice this is minimal in comparison to expected propagation losses.

The phase shifter settings of the binary tree are managed via a National Instruments (NI) Data Acquisition Unit (DAC) with a voltage range between $\pm 10$ V. The $\theta$ phase shifters of the photonic mesh are characterized using a set of monitor taps following the binary tree. Each monitor tap is implemented as a short directional coupler with an approximate 3% coupling strength and a set of focusing grating couplers that redirect the tapped light perpendicular to the chip so that it may be monitored with an overhead infrared camera. To calibrate these $\theta$ phase shifters, the voltage across each heater is sequentially swept while the output of each corresponding monitor tap is recorded. The recorded transmission function is then fitted to the expected intensity functions as determined by Eq. 4. A similar calibration method is implemented for the $\phi$ phase shifters while monitoring the output of the PSGC. This calibration step enables the generation of a mapping between the applied heater voltages and the corresponding phase shifter setting, as well as characterization of the initial phase imbalance of each MZI. Actuation and readout of the calibrated phase shifters enable programmatic generation and analysis of arbitrary polarization states.

The temporal stability of the demonstrated system is dominated primarily by alignment stability in the fiber coupling interface. The path length matching employed throughout the architecture ensures that the operation is nominally independent of global temperature variations, providing a degree of stability against environmental effects. In the present experiments, stable operation is maintained over typical measurement timescales under controlled laboratory conditions. If necessary, slow thermal drifts can be compensated through periodic recalibration of the phase shifters. More generally, programmable photonic meshes based on thermo-optic phase shifters are compatible with active stabilization and self-configuration techniques, which have been widely used to maintain long-term performance^32,43^.

## Data Availability

Data underlying the results presented in this paper are available from the corresponding authors upon request.
